# Magnesium Concentration Modulates Replication Slippage of Mesophilic and Thermophilic DNA Polymerases In Vitro

**DOI:** 10.3390/ijms27156600

**Published:** 2026-07-24

**Authors:** Melissa Castillo-Lizardo, Enrique Viguera

**Affiliations:** 1 German Center for Neurodegenerative Diseases (DZNE), 72076 Tübingen, Germany; melissa.castillo@dzne.de; 2 Área de Genética, Facultad de Ciencias, Campus de Teatinos, Universidad de Málaga, 29071 Málaga, Spain

**Keywords:** replication slippage, DNA polymerase fidelity, magnesium effect, strand displacement activity, direct repeats, hairpin structure

## Abstract

Replication slippage at repetitive DNA sequences generates insertions and deletions that drive genomic instability. Although magnesium ions are essential cofactors for DNA polymerase activity, their role in modulating slippage fidelity remains unclear. Using an *in vitro* primer extension assay on a single-stranded template carrying direct repeats flanking a hairpin-forming inverted repeat, we investigated the effect of Mg^2+^ concentration on slippage produced by mesophilic (T4 Pol, T7 Pol, *E. coli* pol I Klenow fragment, pol I KF exo^−^, and pol III holoenzyme) and thermophilic (*Taq* pol and *Pfu* pol) DNA polymerases. We show that Mg^2+^ modulates slippage frequency in a polymerase-dependent manner, as follows: low concentrations suppress slippage in T7 Pol, pol I KF, pol III HE, and *Taq* Pol, whereas T4 Pol and *Pfu* Pol slip at all productive concentrations. Mechanistically, Mg^2+^ modulates strand displacement activity, and polymerases that acquire enhanced strand displacement at intermediate concentrations show a corresponding reduction in slippage. Proofreading activity had no detectable effect on slippage frequency. These findings reinforce the inverse correlation between strand displacement activity and slippage propensity and suggest that physiological free Mg^2+^ levels may help suppress slippage *in vivo*.

## 1. Introduction

Replication slippage, also termed copy-choice recombination or slipped-strand mispairing, is a mutagenic process that generates insertions and deletions at repetitive DNA sequences. The concept was originally proposed by Streisinger to account for frameshift mutations at homopolymeric runs, where misalignment of the nascent strand at repetitive sequences during DNA synthesis was sufficient to explain the observed mutational spectra [1]. It is now recognised that slippage errors encompass not only single-nucleotide frameshifts but also deletions and insertions between short or long directly repeated sequences in both prokaryotes [2] and eukaryotes [3,4], and the process is considered as a major driver of genomic rearrangements including microsatellite instability [5].

Although mismatch repair systems can correct many of these errors, those that escape correction become fixed mutations. The frequency and nature of slippage events are influenced by the capacity of repetitive sequences to adopt non-B DNA conformations—including hairpins, triplex (H-DNA), and G-quadruplex structures—which stall DNA polymerases and promote misalignment errors [6,7,8]. To elucidate the molecular basis of replication slippage, we developed an experimental system that mimics lagging-strand DNA synthesis across direct repeats (DRs) flanking inverted repeats (IRs) capable of forming a hairpin structure. In this system, the position and spacing of the DRs relative to the IR are fixed, allowing the contribution of secondary structure and repeat position to slippage to be dissected independently of the sequence-context variability inherent to, for example, natural trinucleotide repeat tracts or microsatellites, where both the length of the repeat and the resulting secondary structures can vary considerably. Using this system, we have previously shown that (1) the position of the IR within the DNA template determines the slippage error, since precise stalling of the polymerase within the direct repeat is necessary for slippage error to occur; (2) the length of the direct repeats affects slippage frequency, with shorter repeats favoring slippage, suggesting that only the tip of the new strand is implicated in the realignment of the DR; (3) the kinetics of the reaction show that the polymerase pauses at the base of the hairpin and then dissociates, allowing the 3′ end of the nascent strand to unpair from the first repeat and anneal to the downstream DR, which can lead to deletion of one DR and the intervening IR [9].

We have also investigated the role of the biochemical properties of DNA polymerases in the generation of slippage errors and found an inverse correlation between slippage frequency and the strand displacement activity of a given DNA polymerase [9,10,11,12,13]. DNA polymerases that lack strand displacement activity, such as *Pyrococcus furiosus Pfu* pol, *Escherichia coli* pol II, or T4 pol, generate products lacking one DR and the intervening hairpin as a result of slippage. In contrast, polymerases with high strand displacement activity, such as φ29 DNA polymerase or *Bst* pol, produce high-molecular-weight molecules, indicating that these polymerases can unwind and replicate through the hairpin structure, thereby preventing slippage errors [11,12,13].

Beyond the intrinsic properties of the polymerase, several biochemical parameters modulate slippage frequency *in vitro*. SSB modulates slippage in a concentration-dependent manner—at sub-saturating concentrations, it enhances processivity and increases slippage, whereas at saturating concentrations, it suppresses slippage by stimulating strand displacement activity, enabling the polymerase to traverse the hairpin. Crucially, SSB acts by modulating strand displacement rather than by melting template secondary structures [11]. The effect of Mg^2+^ concentration on slippage is polymerase dependent, as follows: for polymerases with intermediate strand displacement activity, such as *Taq* pol and Vent^+^ pol, increasing Mg^2+^ progressively favours slippage; however, for polymerases that slip regardless of conditions, such as *Pfu* pol and *Pab* polB exo^−^ (Pyra exo^−^), Mg^2+^ concentration has no modulatory effect on slippage frequency [12].

In order to study the role of Mg^2+^ concentration on the slippage error produced by thermolabile and thermophilic DNA polymerases under different optimal reaction temperatures, we have conducted primer extension reactions on the ssDNA template containing two direct repeats (DRs) flanking inverted repeats (IRs) capable of forming a hairpin structure.

## 2. Results

### 2.1. Effect of Magnesium Concentration on the Replication Slippage of Mesophilic DNA Polymerases

**Figure 1:**
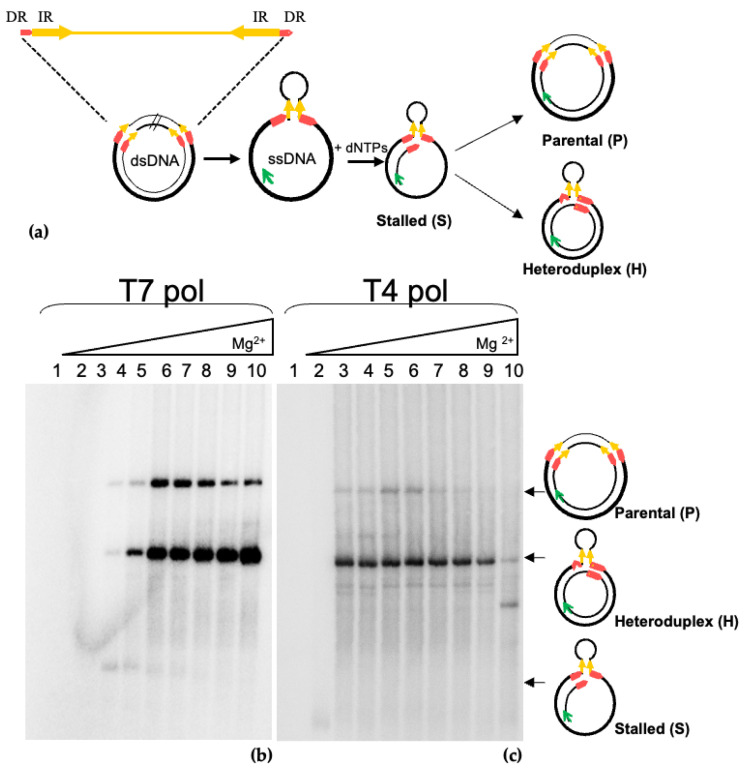
Effect of magnesium concentration on slippage of DNA polymerases T7 and T4. Schematic representation of the double-stranded plasmid pHP27FXc that contains two 27-bp direct repeats (DR, red arrows) flanking two 300-bp inverted repeats (IR, yellow arrows). The single-stranded DNA template (ssDNA) is obtained *in vitro*. Primer extension reactions were carried out on the primed-ssDNA-FXc template at 37 °C (1233 primer, green arrow) (**a**). Reactions contained 0.5 units of T7 DNA polymerase (**b**) and 3 units T4 DNA polymerase (**c**), 75 ng of SSB, and increasing concentrations of MgCl_2_. Lanes 1–10: 0, 0.1, 0.5, 1, 2.5, 5, 7.5, 10, 15 and 20 mM MgCl_2_. P, H, and S refer to parental, heteroduplex, and stalled molecules, respectively.

The schematic representation of the DNA template used is shown in Figure 1a. The replication slippage of DNA polymerases from phages T4 and T7 was analysed at increasing magnesium concentrations ranging from 0 to 20 mM (Figure 1b,c). Two effects were observed, one on total DNA synthesis and the other on slippage frequency. At very low magnesium concentrations (0.1 mM), there was essentially no synthesis (Figure 1b,c, lane 2), while at the highest concentration tested (20 mM), synthesis by T4 pol was significantly inhibited (Figure 1c, lane 10). For T7 pol, low magnesium concentrations (1 mM) produced stalled molecules arrested at the base of the hairpin, together with some heteroduplex products (Figure 1b, lane 4). At intermediate magnesium concentrations (2.5–10 mM), heteroduplex products in addition to faithful replicated parental molecules were generated (Figure 1b, lanes 5–8). At high magnesium concentrations (15–20 mM), heteroduplex molecules generated by slippage became the predominant reaction products (Figure 1b, lanes 9–10). In contrast, T4 pol synthesised predominantly heteroduplex molecules whenever synthesis was efficient, independently of magnesium concentration (Figure 1c, lanes 3–9).

**Figure 2:**
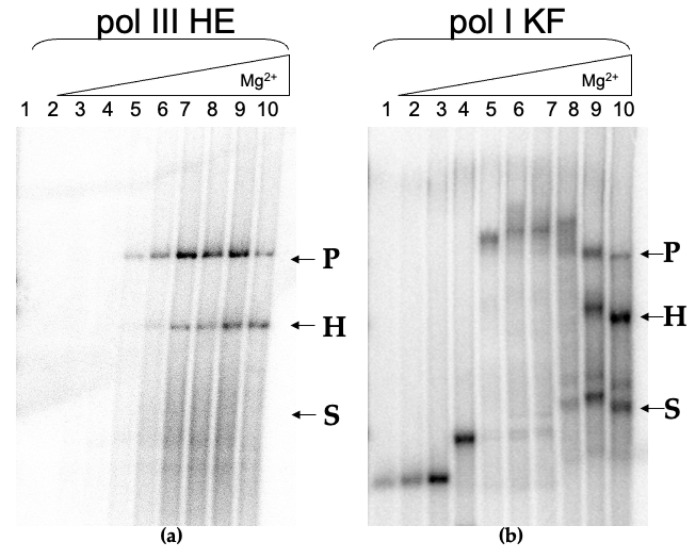
(**a**) Effect of magnesium concentration on slippage of DNA polymerase pol III HE. (**b**) Effect of magnesium concentration on slippage of pol I Klenow fragment. Primer extension reactions were carried out as on FXc template at 37 °C. Reactions contained 0.5 µL of pol III HE and 1 unit of pol I Klenow fragment and increasing concentrations of MgCl_2_. Lanes 1–10: 0, 0.1, 0.5, 1, 2.5, 5, 7.5, 10, 15 and 20 mM MgCl_2_. P, H, and S refer to parental, heteroduplex, and stalled molecules, respectively.

Previous work demonstrated that *E. coli* DNA polymerase I Klenow fragment (pol I KF) and DNA polymerase III holoenzyme (pol III HE) slip between direct repeats [10,11]. Here we investigated the effect of varying magnesium concentration on slippage produced by these two polymerases. Results are showed in Figure 2. For pol III HE, the results were similar to those obtained with T7 pol, as follows: at the lowest magnesium concentrations (0.1–1 mM) there was essentially no synthesis (Figure 2a, lanes 1–4); at intermediate concentrations (2.5–15 mM), both parental and heteroduplex molecules were generated, with parental molecules predominating (Figure 2a, lanes 5–9); and at the highest magnesium concentration, pol III HE generated mainly heteroduplex molecules (Figure 2a, lane 10).

For pol I KF, low magnesium concentrations yielded no or very little synthesis, with stalled molecules visible at the lowest productive concentration (Figure 2b, lanes 1–4). Strikingly, at intermediate magnesium concentrations, pol I KF produced molecules migrating more slowly than the parental duplex, indicating a higher molecular weight (Figure 2b, lanes 5–8). This result is consistent with rolling circle replication, arising when, after completion of one round of replication, the newly synthesised strand is displaced and synthesis continues. At the highest magnesium concentrations, slippage was promoted and heteroduplex molecules became the main products (Figure 2b, lanes 9–10). Taken together, these results demonstrate that the mesophilic polymerases T4 pol, T7 pol, *E. coli* pol I KF, and pol III HE slip differentially depending on magnesium concentration.

### 2.2. Effect of Magnesium Concentration on Slippage Errors of the Proofreading-Deficient Mutant Pol I KF Exo^−^

**Figure 3:**
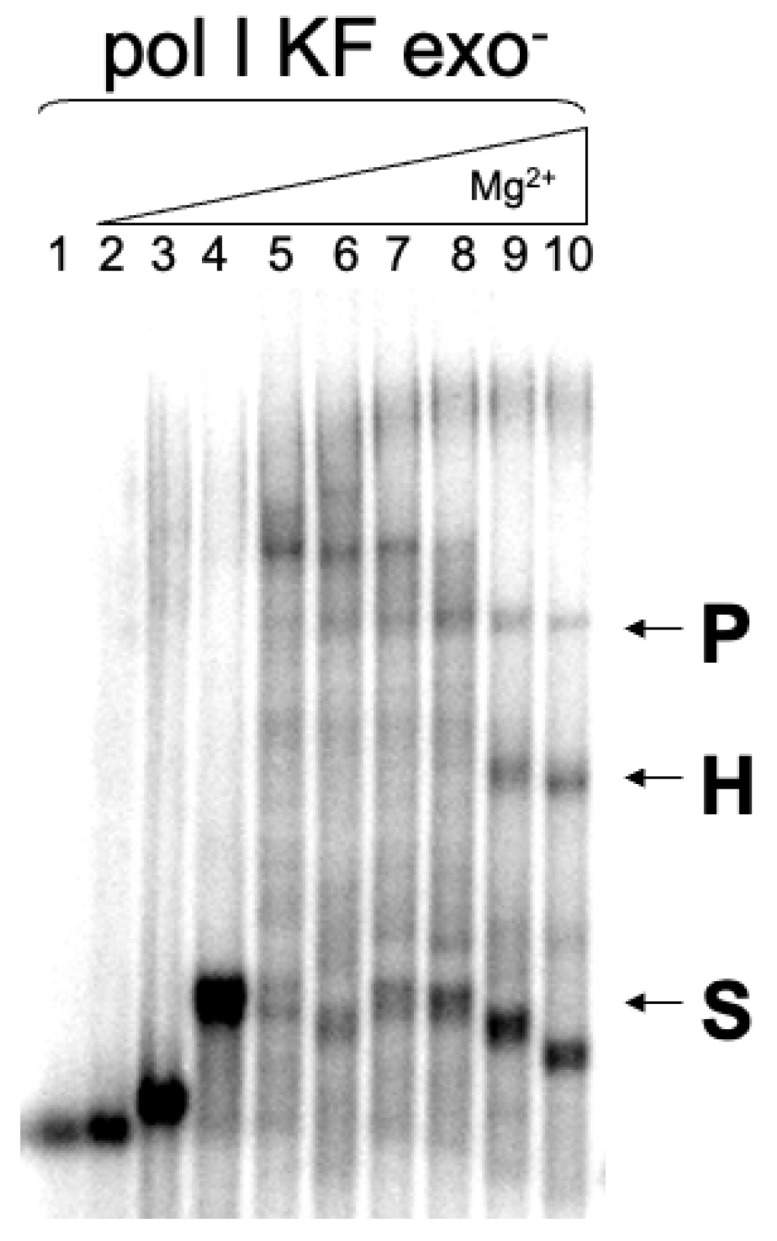
Effect of magnesium concentration on slippage of DNA polymerase pol I klenow fragment exo^−^. Primer extension reactions were carried out on FXc template at 37 °C. Reactions contained 1 unit of pol I Klenow fragment exo^−^, 75 ng of SSB and increasing concentrations of MgCl_2_. Lanes 1–10: 0, 0.1, 0.5, 1, 2.5, 5, 7.5, 10, 15 and 20 mM MgCl_2_. P, H, and S refer to parental, heteroduplex, and stalled molecules, respectively.

To study the effect of proofreading activity on slippage at increasing magnesium concentrations, the exonuclease-deficient form of pol I KF (pol I KF exo^−^) was tested. The results are presented in Figure 3. The slippage behaviour of pol I KF exo^−^ was not very different from that of the wild-type enzyme with respect to the slippage error (compare Figure 2b and Figure 3), indicating that the 3′ → 5′ proofreading exonuclease activity does not substantially influence slippage frequency at the magnesium concentration tested. A higher quantity of stalled molecules was found for pol I KF exo^−^, which suggests that this enzyme is more prone to stalling upon encountering the hairpin region.

### 2.3. Effect of Magnesium on the Replication Slippage of Thermophilic DNA Polymerases

*Taq* and *Pfu* DNA polymerases are widely used in molecular biology, for routine amplification and high-fidelity applications, respectively. These thermophilic DNA polymerases are able to slip at repeated sequences [12]. Here we investigated the effect of magnesium concentration on the fidelity of replication at repeated sequences for these polymerases on native agarose gel electrophoresis and strand displacement activity on acrylamide-urea sequencing gel.

**Figure 4:**
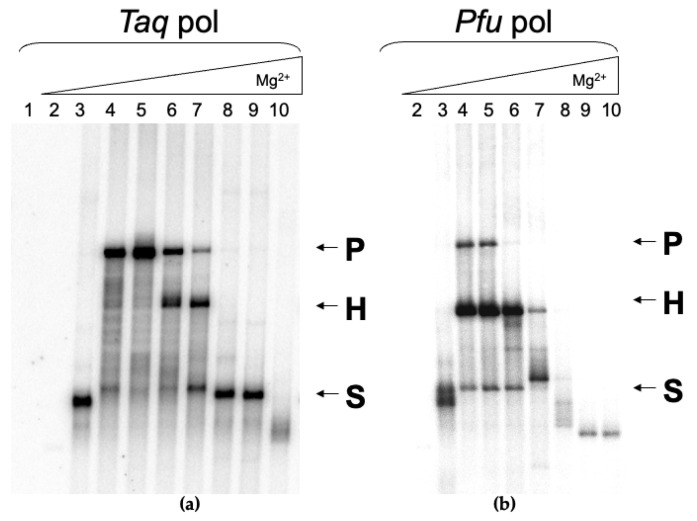
(**a**) Effect of magnesium concentration on slippage of *Taq* DNA polymerase. (**b**) Effect of magnesium concentration on slippage of *Pfu* DNA polymerase. Primer extension reactions were carried out on template FXc at 60 °C. Reactions contained 0.1 and 0.5 units of *Taq* and *Pfu* DNA polymerases, respectively, and increasing concentrations of MgCl_2_. Lanes 1–10: 0, 0.1, 0.5, 1, 2.5, 5, 7.5, 10, 15 and 20 mM MgCl_2_. P, H, and S refer to parental, heteroduplex, and stalled molecules, respectively.

The results shown in Figure 4a indicate that at low magnesium concentrations (1–2.5 mM), *Taq* pol showed the highest fidelity in terms of slippage, as the only detected products were fully replicated parental molecules (Figure 4a, lanes 4–5), indicating that the polymerase was able to progress through the hairpin under these conditions. At intermediate magnesium concentrations (5–7.5 mM), heteroduplex molecules were detected, revealing that *Taq* pol was no longer able to enter the hairpin and thereby generated deletion products (Figure 4a, lanes 6–7). At the highest magnesium concentrations tested (10–20 mM), stalled molecules predominated, consistent with arrest of the polymerase at the base of the hairpin (Figure 4a, lanes 8–10). In contrast, *Pfu* pol behaved very differently, as follows: heteroduplex molecules were the predominant product at all productive magnesium concentrations tested, with some stalled molecules also detected (Figure 4b, lanes 4–8), indicating that unlike *Taq* pol, the fidelity of *Pfu* pol at repeated sequences is not rescued at low magnesium concentrations. At the highest magnesium concentration, synthesis was inefficient (Figure 4b, lanes 9, 10).

These results demonstrate that the fidelity of *Taq* pol, in terms of slippage, is sensitive to magnesium concentration. In contrast, *Pfu* pol is very prone to slip at all magnesium concentrations tested, whenever synthesis is efficient.

### 2.4. Effect of Magnesium Concentration on the Strand Displacement Activity of T4, T7, Pol I Klenow Fragment, and Pol I Klenow Fragment Exo^−^ DNA Polymerases

An inverse correlation between strand displacement activity and slippage has been established in our previous work [11,12]. Moreover, we have demonstrated that factors acting in trans, such as *E. coli* SSB, are able to stimulate the strand displacement activity of T7 DNA polymerase [11]. Taken together, this evidence prompted us to investigate whether magnesium concentration modulates the strand displacement activity of the DNA polymerases under study.

To test this hypothesis, we used a previously established assay under the same conditions used for studying the effect of magnesium concentration on slippage [11]. The system consists of a double-primed M13mp18 ssDNA template, in which one primer 17 bp homologous to the template is 5′-end-labelled and a second primer, placed 75 nucleotides downstream, is non-homologous to the template over its 5′-terminal 10 bases and annealed over the remaining 20 bases. Replication products from the labelled primer are analysed on 6% denaturing sequencing gels. A polymerase lacking strand displacement activity will stall upon encountering the second primer, generating a labelled fragment of 91 nucleotides. In contrast, a polymerase endowed with strand displacement activity will synthesise through the double-stranded region and generate fragments longer than 91 nucleotides.

**Figure 5:**
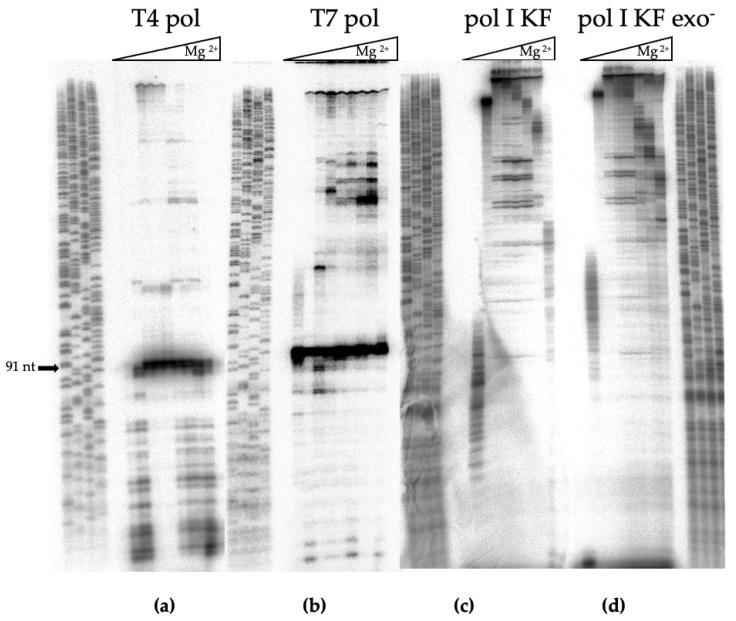
(**a**) Effect of magnesium concentration on strand displacement activity of DNA polymerases T4, (**b**) T7, (**c**) pol I klenow fragment, and (**d**) pol I Klenow fragment exo^−^. Primer extension reactions were carried out on ssM13mp18 template at 37 °C. Reactions contained 0.5 units of each polymerase, 75 ng of SSB and increasing concentrations of MgCl_2_: 0, 0.1, 0.5, 1, 2.5, 5, 7.5, 10, 15 and 20 mM (Lines 1–10). A sequencing ladder of ssM13mp18 obtained with the −40 primer was loaded on each gel. The arrow labelled 91 nt indicates the position of the labelled fragment that results from replication of ssDNA from the labelled primer −40 up to the downstream primer.

The measurement of strand displacement activity of T4 pol revealed products of 91 nucleotides at all magnesium concentrations tested, demonstrating the absence of strand displacement activity in this polymerase (Figure 5a). In contrast, T7 pol exhibited moderate strand displacement activity at intermediate magnesium concentrations, as deduced from the presence of molecules longer than 91 nt, with this activity diminishing at both the lowest and highest concentrations tested (Figure 5b). Pol I KF produced high-molecular-weight products predominantly at intermediate magnesium concentrations, consistent with high strand displacement activity under these conditions (Figure 5c). No significant differences were found for pol I Klenow fragment exo^−^ (Figure 5d).

**Figure 6:**
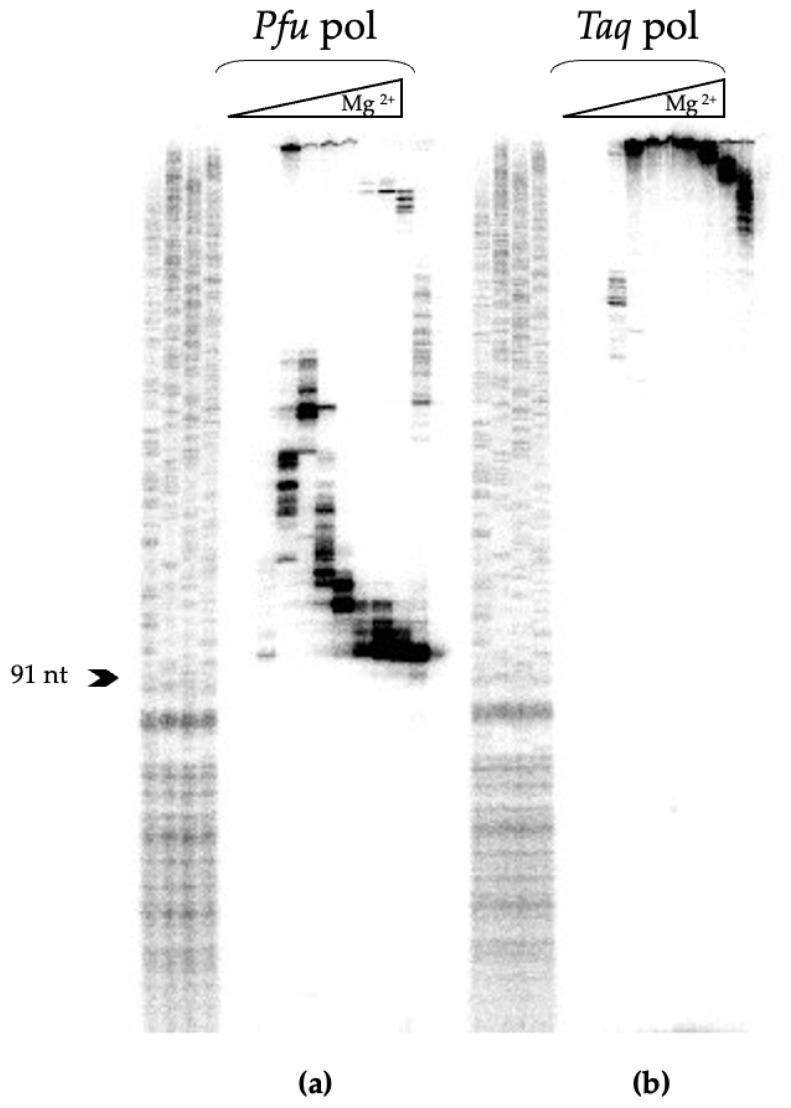
(**a**) Effect of magnesium concentration on strand displacement activity of *Pfu* and (**b**) *Taq* DNA polymerases. Primer extension reactions were carried out on ssM13mp18 template at 60 °C. Reactions contained 0.5 units of each polymerase and increasing concentrations of MgCl_2_: 0, 0.1, 0.5, 1, 2.5, 5, 7.5, 10, 15 and 20 mM (Lines 1–10). A sequencing ladder of ssM13mp18 obtained with the −40 primer was loaded on each gel. The arrow labelled 91 nt indicates the position of the labelled fragment that results from replication of ssDNA from the labelled primer −40 up to the downstream primer.

Furthermore, *Pfu* pol showed moderate strand displacement activity, stimulated at intermediate magnesium concentrations and reduced at both the lowest and highest concentrations tested (Figure 6a). In contrast, *Taq* pol was characterised by high strand displacement activity predominantly at low-to-intermediate magnesium concentrations, which diminished at the highest concentrations tested (Figure 6b). Taken together, these results reveal a strong correlation between strand displacement activity and slippage frequency. Previous studies have shown that polymerases endowed with high strand displacement activity do not slip at repeated sequences [11,12]. The present data extend this finding by demonstrating that magnesium concentration modulates strand displacement activity in polymerases that possess some intrinsic capacity for strand displacement, thereby providing a mechanistic basis for the magnesium-dependent modulation of slippage observed in these enzymes.

## 3. Discussion

Magnesium concentration affects the fidelity of replication at two levels. First, it directly modulates polymerase activity and fidelity [14,15]; second, it influences DNA duplex stability, thereby affecting the capacity of repetitive sequences to form secondary structures that promote slippage [16,17]. Since both of these parameters are expected to influence slippage frequency, we studied the effect of varying magnesium concentration on the fidelity of replication at repeated DNA sequences on a single-stranded DNA template carrying a hairpin structure by performing primer extension reactions with mesophilic (pol III HE, pol I KF, pol I KF exo^−^, T7 pol, and T4 pol) and thermophilic polymerases (*Taq* pol and *Pfu* pol).

A summary of the results is showed in Table 1. The results showed that replication slippage of both mesophilic and thermophilic DNA polymerases is dependent on the magnesium concentration, and that this effect is not due to overall inhibition of synthesis. Low-Intermediate magnesium concentration inhibits slippage produced by pol III HE, pol I KF, pol I KF exo^−^, and T7 pol. However, T4 pol generates slippage error at all the magnesium concentrations tested provided that synthesis is efficient. Interestingly, a previous study investigated the effects of sodium chloride and potassium glutamate on slippage of pol III HE and found that low concentrations of these salts stimulated slippage [11], indicating that ionic conditions modulate slippage frequency. The intracellular magnesium concentration in *E. coli* is estimated to be around 100 mM [18]; however, free magnesium is only approximately 1–5 mM [19], suggesting that at physiological magnesium concentrations, slippage may be inhibited *in vivo*.

Regarding the thermophilic polymerases tested, low magnesium concentration inhibits slippage produced by *Taq* pol as parental molecules were mainly obtained. However, native *Pfu* pol generates heteroduplex products at intermediate magnesium concentration. Only a very minor fraction of parental molecules were detected for *Pfu* pol at 1 and 2.5 mM MgCl_2_, in contrast to our previously reported results, in which only heteroduplex molecules were detected at the equivalent magnesium concentration [12]. As this enzyme was obtained commercially rather than purified in-house, and the manufacturer does not disclose details that would allow direct comparison with the previously used preparation, we cannot rule out that a difference in formulation or a proprietary modification accounts for this discrepancy.

An inverse correlation between the strand displacement activity of a DNA polymerase and its ability to slip was previously established [11,12]. In the experimental system used in this work, the strand displacement activity of a polymerase determines its ability to progress through the hairpin and thus generate a fully replicated parental molecule. Conversely, a polymerase lacking strand displacement activity may pause at the base of the hairpin, dissociate, and allow the newly synthesised strand to anneal with the second direct repeat, resume replication, and ultimately generate a heteroduplex molecule [9]. Therefore, factors that stimulate strand displacement activity are expected to modulate slippage frequency.

Magnesium concentration also affects the strand displacement activity of T7 pol and *Taq* pol as an increased strand displacement activity was observed at intermediate magnesium concentrations (Figure 5b and Figure 6b). These results correlate with the presence of parental molecules in the primer extension assays on the hairpin-containing template (Figure 1b and Figure 4a). In contrast, T4 pol did not show strand displacement activity at any of the magnesium concentrations tested (Figure 5a). This result explains that only heteroduplex molecules were found for T4 pol (Figure 1c). Notably, *Pfu* pol shows some strand displacement activity at intermediate magnesium concentrations (Figure 6a), which explains the minor fraction of parental molecules detected (Figure 4b). Finally, pol I KF is endowed with a very high strand displacement activity (Figure 5c) [20,21], and consequently high-molecular-weight molecules were found at intermediate magnesium concentrations; only a minor fraction of heteroduplex molecules were found at the highest magnesium concentration tested (Figure 2b). Owczarzy et al. (2008) [16] demonstrated that magnesium ions stabilise DNA duplexes in a concentration-dependent and non-linear manner, with stability reaching a maximum at intermediate concentrations before declining at higher ones. Since hairpin stability is a critical determinant of polymerase stalling in our system, progressive stabilisation of the IR hairpin with increasing magnesium concentration would be expected to impair strand displacement and promote slippage, consistent with our findings.

Proofreading has an important role in replication fidelity, improving average accuracy approximately 10-fold [22]. We found no effect of proofreading activity on slippage efficiency of pol I KF exo^−^ under our experimental conditions (Figure 2b and Figure 3). These results are consistent with findings that exonuclease activity does not increase the fidelity of synthesis at dinucleotide repeat sequences during PCR [23]. When misalignments occur at repeated sequences, the unpaired bases can be positioned outside the polymerase active site and correct base pairs can stabilise the misalignment [24]. Repeated sequences thus provide a stable misaligned intermediate that promotes continued polymerisation rather than exonucleolytic correction, explaining why frameshift errors frequently escape exonucleolytic correction [24,25]. Ramanathan et al. (2002) [26] investigated the effect of Klenow exo^+^ and exo^−^ enzymes on the replication of a G–G:C triplex and found that the exonuclease deficient Klenow is able to replicate through the G–G:C triplex structure with an efficiency inversely dependent on magnesium concentration, whereas Klenow exo^+^ cannot. This contrasts with our results, where increasing magnesium generally promoted slippage in polymerases with intermediate strand displacement activity on a canonical Watson–Crick IR hairpin. Additionally, whereas Ramanathan et al. [26] found a dramatic difference between exo^+^ and exo^−^ Klenow in traversing the G–G road-block, we observed no significant effect of proofreading activity on slippage frequency or its modulation by magnesium. These differences could be explained by the nature of the template secondary structure, as resolution of a G–G:C triplex depends critically on triplex stability, whereas slippage at a Watson–Crick IR hairpin depends on the strand displacement activity of a polymerase, which is modulated by magnesium concentration.

Previous observations have suggested a positive correlation between processivity and the fidelity of DNA polymerases [27], a correlation first proposed on the basis that polymerase α is both more accurate and more processive than polymerase β [28]. Because the molecular mechanism of slippage involves polymerase pausing and dissociation [9], polymerases with high processivity would be expected to slip less frequently. However, our findings do not reveal a correlation between processivity and slippage on hairpin-containing templates. Pol III HE, a highly processive complex when associated with its accessory proteins [29], can slip, whereas pol I KF, which has low processivity [30,31], shows high fidelity for slippage. This is consistent with observations that SSB and the β subunit, both of which enhance the processivity of pol III HE, promote rather than suppress slippage of the complex [10]. Supporting this, decreased processivity of *Taq* pol did not significantly affect the generation of frameshift products, and T4 pol shows low fidelity for deletions in homonucleotide runs even when synthesising DNA in a highly processive manner with its replication accessory proteins [23,32]. Thus, while greater processivity may in general contribute to improved fidelity, this does not appear to be the case for slippage errors under our experimental conditions.

Interestingly, the folded replication slippage model recently proposed by Zhang et al., in which template strand folding angle dictates hydrogen-bond disruption and thereby repeat expansion versus contraction [33], raises the possibility that Mg^2+^-dependent changes in duplex stability could shift this folding equilibrium—a hypothesis that resolving the molecular mechanism by which Mg^2+^ concentration influences DNA secondary structure stability and polymerase behaviour at the single-molecule level would help test. However, addressing this question would require single-molecule approaches capable of directly following polymerase progression in real time, which lies beyond the scope of the bulk biochemical assays used in this study.

**Figure 7:**
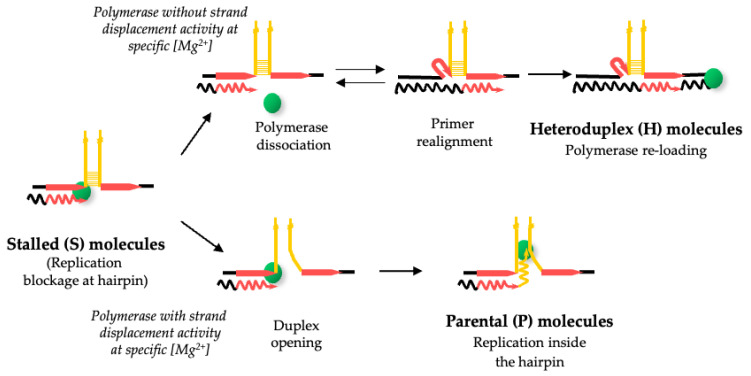
Model proposed for replication slippage between direct repeats promoted by hairpin-containing templates. Diagram shows part of a single-stranded template (straight lines) with newly synthesized DNA (wavy lines) and the DNA polymerase (green sphere). Direct repeats (red arrows) flank inverted repeats (yellow) that anneal to form a hairpin structure. Slippage-mediated deletion is proposed to involve three steps (Top): (1) the polymerase pauses within the first direct repeat at the base of the hairpin, (2) polymerase dissociation, and (3) strand misalignment, where the 3′ end of the nascent strand unpairs from the complementary strand and reanneals with the second direct repeat, thus generating a deletion [9]. However, a polymerase with high strand displacement activity is able to open the hairpin duplex (bottom) and replicate the repeat-containing template faithfully. Depending on the strand displacement activity of the DNA polymerase and specific magnesium concentration, the reaction may generate stalled molecules (S), heteroduplex molecules (H), or parental molecules (P). Blockage of the nascent strand before reaching the hairpin generates short primer/template molecules of different lengths. Blockage of the nascent strand within the hairpin generates stalled molecules of different lengths that cannot progress to form heteroduplex molecules, as the 3′ end is not complementary to the second direct repeat.

In conclusion, we propose that magnesium promotes replication fidelity at repeated DNA sequences by modulating strand displacement activity, reinforcing the importance of the inverse correlation between the strand displacement activity of a polymerase and its propensity to slip [11,12], Figure 7. These results contribute to our understanding of the dynamics of replication slippage, though a key outstanding question concerns the relationship between observations made with purified polymerases *in vitro* and DNA replication *in vivo*.

## 4. Materials and Methods

### 4.1. Proteins

T4 pol, T7 pol, pol I KF, and pol I KF exo^−^ were purchased from New England Biolabs, Ipswich, MA, USA. *Taq* pol was from Roche Molecular Biochemicals, Mannheim, Germany, and Native *Pfu* pol was from Stratagene, La Jolla, CA, USA. Reconstituted pol III* (holoenzyme minus the β subunit) and the β subunit were a kind gift of Prof. Charles McHenry (University of Colorado). Sequencing was carried out according to the protocol of the Sequenase version 2 sequencing kit (U. S. Biochemical Corp., Cleveland, OH, USA). *E. coli* SSB was purchased from Promega, Madison, WI, USA. Proteinase K was from Roche Molecular Biochemicals. M13 gene protein II (gp II) was purified to homogeneity as described [9].

### 4.2. Chemicals

#### 4.2.1. ssDNA and Primer-Extension Reactions

[γ-^32^P]dATP (3000 Ci/mmol) and [α-^32^P]dCTP (3000 Ci/mmol) were purchased from PerkinElmer, Waltham, MA, USA. Unlabelled nucleotides were from Amersham Pharmacia Biotech, Little Chalfont, Buckinghamshire, UK. Plasmid pHP727FXc DNA was extracted using a Maxi Plasmid Kit followed by using a QIAquick^®^ PCR purification kit, and both were purchased from Qiagen, Hilden, Germany. pHP727FXc ssDNA template was obtained after gpII nick of the 6.128 bp pHP727FXc dsDNA and exonuclease III digestion of the nicked strand as described [9]. The primer extension reactions were performed as described [9,12]. Briefly, a primer designated #1233 (5′ AGC GGA TAA CAA TTT CAC ACA GGA 3′) was annealed 1235 bases from the palindrome. All primer extension reactions contained in 10 µL: 25 ng of primed ssDNA, 200 µM dNTP (each) if ^32^P-labelled primer was used, or 200 µM dGTP, dATP, and dTTP (each) and 50 µM (2.5 mCi) [α-^32^P]dCTP. SSB and each DNA polymerase were added to the reaction mixture as indicated in the legends of the figures. Reactions were preincubated 5 min at 37 °C in the presence or in the absence of SSB, with all the other components, before DNA polymerase addition. The reaction buffers were prepared magnesium free as those furnished by the suppliers and contained, in addition to 30 mM NaCl brought by the primed ssDNA, the following ingredients: (i) for pol III HE: 33 units (75 ng) Pol III*, 10 ng β subunit in 20 mM Tris-HCl (pH 7.5), 2 mM DTT, 2 mM ATP, 100 µg of BSA per ml, 10% glycerol; (ii) for T4 pol, pol I KF, pol I KF exo^−^: 50 mM NaCl, 10 mM Tris-HCl, pH 7.9, 1 mM DTT; (iii) for T7 pol: 20 mM Tris-HCl, pH 7.5, 1 mM DTT; (iv) for *Taq* pol: 10 mM Tris-HCl, pH 8.3, 50 mM KCl; (v) for Native *Pfu* pol: 20 mM Tris-HCl, pH 8.0, 10 mM KCl, 6 mM (NH4)_2_SO_4_, 0.1% Triton^®^ X-100, 10 µg/mL BSA. MgCl_2_ was added to a final concentration of 0.1–20 mM as indicated in figure legends. After 15 min at 37 °C (thermolabile polymerases) or 60 °C (thermophilic polymerases), the synthesis was arrested by the addition of 25 mM EDTA and 500 µg/mL proteinase K, and the mixture was further incubated for 15 min at 55 °C. Reaction products were analyzed by electrophoresis through 0.8% agarose gels under native conditions, run in TBE buffer (89 mM Tris-borate, 2 mM EDTA, pH 8.3) at 2 V/cm for 16 h. DNA was visualized by direct exposure of the dried gels either to X-ray films or to Imaging Plates (IP BAS-MP 2040S) and analyzed on a Fujifilm-BAS 1500 (Fujifilm Holdings Corporation, Tokyo, Japan). Each experiment was performed independently 2–3 times, depending on the specific assay, except for the reactions performed with Pol III HE, which were carried out once due to the limited amount of purified enzyme available. Minor variation in band intensity was observed across these independent replicates, reflecting a somewhat higher or lower proportion of the different reaction products, but no qualitative differences in the pattern of products obtained. The gel images shown throughout the manuscript are representative of these independent experimental replicates. Polymerase activity was tested in an independent replication assay with the purified DNA polymerase on the circular M13mp18 ssDNA under similar Mg^2+^ conditions as used on the replication assays on the FXc template. This control allowed us to determine that synthesis efficiency per se is not impaired across the tested magnesium range (Supplementary Figure S1). A standard molecular weight marker was not included in these gels, as the parental, heteroduplex, and stalled species differ in DNA topology (circular, hairpin-containing, or partially single-stranded) and therefore do not migrate according to a standard linear DNA size marker. The FXc template used in this study is the same as that previously characterised in our earlier work [12], where the identity and size of the P, H, and S products were established by restriction digestion with the enzymes *Xmn*I and *Rsa*I, followed by determination of fragment sizes by polyacrylamide gel electrophoresis. We have added as migration control a sample consisting of the product of a replication assay on the FXc template containing parental, heteroduplex, or stalled molecules that we have previously characterised by restriction analysis in an independent experiment (see Supplementary Materials File S2, uncropped images). The identity of the bands in the present study is therefore based on their migration pattern relative to this previously established characterisation.

#### 4.2.2. Measurement of Strand Displacement Activity

M13mp18 ssDNA was from New England Biolabs. The following two primers were used: 1212 (5′ GTT TTC CCA GTC ACG ACG TTG TA 3′) and 37 (5′ CTA ATC AGG AGA ATT CGT AAT CAT GGT CAT 3′). Primer 1212 was labelled and annealed using a 2-fold molar excess at positions 6326–6310 of ssM13mp18. Primer 37 was annealed using a 2-fold molar excess at positions 6235–6216 (only 20 bases from the 3′ end are complementary to the template, whereas 10 bases form a 5′ tail). Primer extension reactions were performed as described above, except that they contained 25 ng of doubly primed single stranded M13mp18. Products were analyzed by electrophoresis through a 6% acrylamide-urea sequencing gel, run at 60 watts for 90 min. DNA was visualised as described above.

## Figures and Tables

**Table 1:** Effect of magnesium concentration on slippage error on FXc template of T7 pol, T4 pol, Pol III HE, pol I KF, pol I KF exo^−^, *Taq* Pol, *Pfu* pol native (This work) and *Pfu* pol [9].

DNA Polymerase ^1^	Magnesium Concentration (mM)								
	0.1	0.5	1	2.5	5	7.5	10	15	20
T7 pol	-	-	P/H/S	P/**H**	P/**H**	P/**H**	P/**H**	P/**H**	P/**H**
T4 pol	-	-	**H**	**H**	**H**	**H**	**H**	**H**	H/S
pol III HE	-	-	-	**P**	**P**	**P**/H	**P**/H	**P**/H	**P/H**
pol I KF	p/t	p/t	**S**	**rcr**	**rcr**	**rcr**	**rcr**	P/H/S	P/**H/S**
polIKF exo^−^	p/t	**S**	**S**	**rcr**	**rcr**	**rcr**	**rcr**	P/H/**S**	P/H/**S**
*Taq* Pol	-	**S**	**P**	**P**	**P/H**	P/**H**/S	**S**	**S**	**S**
*Pfu* Pol (native)	-	**S**	P/**H**	P/**H**	**H**	H/**S**	**S**	p/t	p/t
*Pfu* Pol	**S**	**H**	**H**	**H**	**H**	**H**	**S**	**S**	nd

^1^ The main product obtained for each reaction is shown in bold. H indicates heteroduplex molecules, generated by replication slippage error. P indicates parental molecules, indicative of faithful replication. S indicates stalled molecules arrested at the base of the hairpin. rcr indicates high-molecular-weight molecules generated by rolling circle replication because of the strand displacement activity of a DNA polymerase. p/t: primer-template or incomplete extension events. nd: not determined.

## Data Availability

The original contributions presented in this study are included in the article/Supplementary Materials. Further inquiries can be directed to the corresponding author.

## Supplementary Materials

The following supporting information can be downloaded at: https://www.mdpi.com/article/10.3390/ijms27156600/s1.
