# Supplementary material for: Magnesium Concentration Modulates Replication Slippage of Mesophilic and Thermophilic DNA Polymerases In Vitro
Source: Int J Mol Sci. 2026 Jul 24;27(15):6600. doi: 10.3390/ijms27156600 (PMC13465473; doi:10.3390/ijms27156600)
Supplement: Supplementary file 1 [file ijms-27-06600-s001.zip › File S1-Figure S1.pdf]

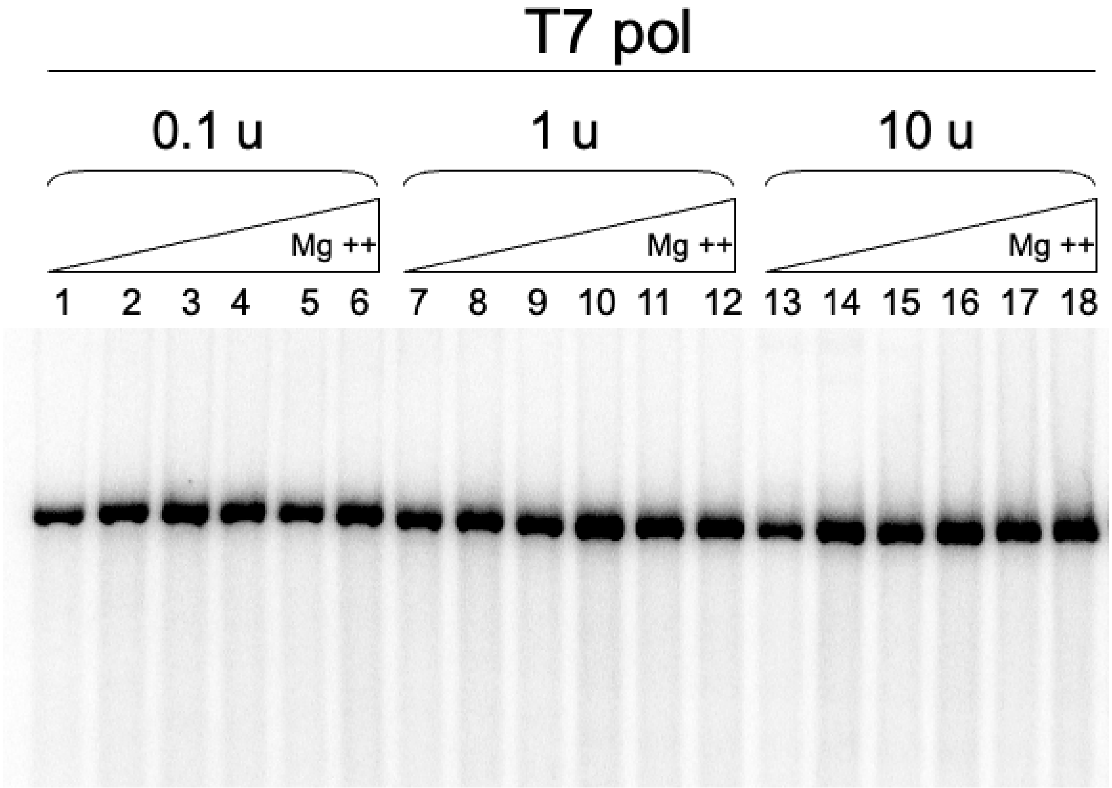

Figure S1. Effect of magnesium concentration on synthesis efficiency during primer extension by T7 DNA polymerase. Primer extension reactions were carried out as described in Materials and Methods, on 25 ng of ssM13mp18 template at 37ºC. Reactions contained 37.5 ng of SSB and increasing concentrations of $MgCl_2$. Lanes 1-6 (0.1 units T7 pol), 7-12 (1 units T7 pol) and 13-18 (10 units T7 pol): 2.5, 5, 7.5, 10, 15 and 20 mM $MgCl_2$.
