# Supplementary material for: Magnesium Concentration Modulates Replication Slippage of Mesophilic and Thermophilic DNA Polymerases In Vitro
Source: Int J Mol Sci. 2026 Jul 24;27(15):6600. doi: 10.3390/ijms27156600 (PMC13465473; doi:10.3390/ijms27156600)
Supplement: Supplementary file 1 [file ijms-27-06600-s001.zip › File S2.pdf]

Magnesium concentration modulates replication slippage of mesophilic and thermophilic DNA polymerases in vitro

Melissa Castillo-Lizardo and Enrique Viguera*

* Correspondence: eviguera@uma.es

Original uncropped gels

Figure 1. Effect of magnesium concentration on slippage of DNA polymerases T7 and T4

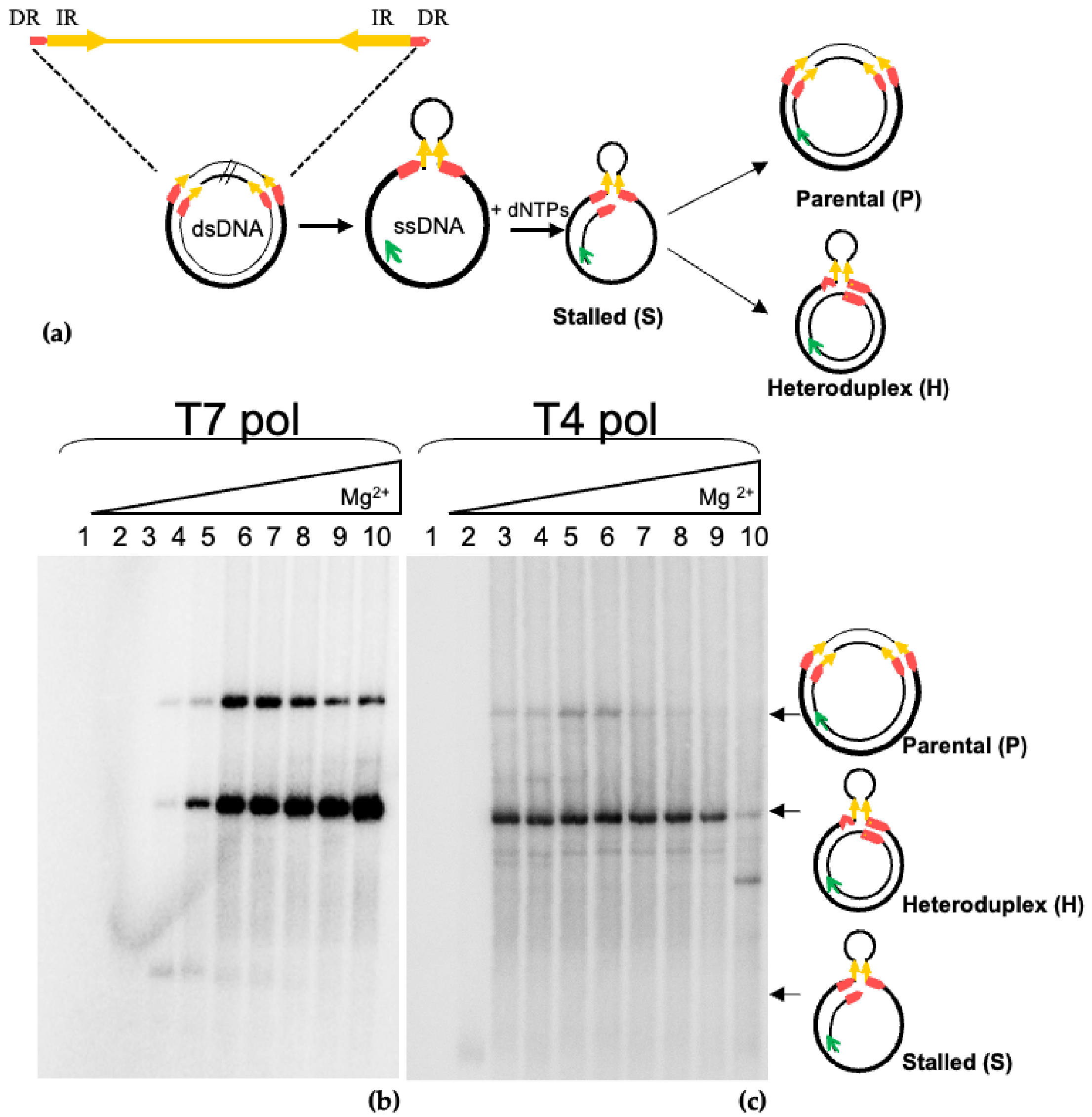

Uncropped gels

Migration control

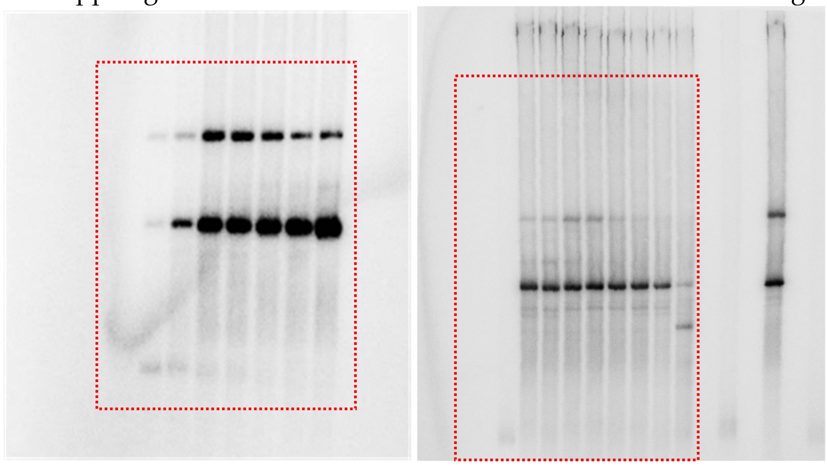

Figure 1 (b) **Figure 1 (c)**

Magnesium concentration modulates replication slippage of mesophilic and thermophilic DNA polymerases in vitro Castillo-Lizardo, Melissa and Enrique Viguera. Universidad de Málaga.

Figure 2. (a) Effect of magnesium concentration on slippage of DNA polymerase pol III HE. (b) Effect of magnesium concentration on slippage of pol I Klenow fragment.

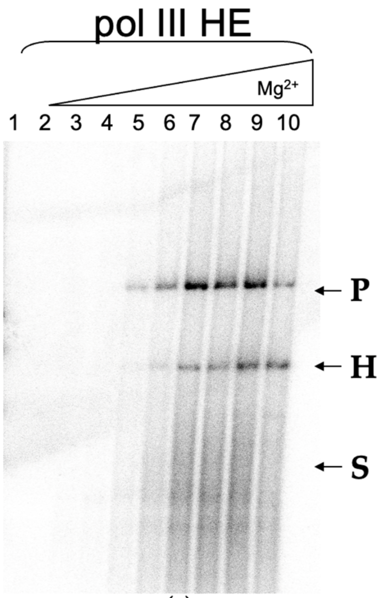

(a)

Migration control Uncropped gel

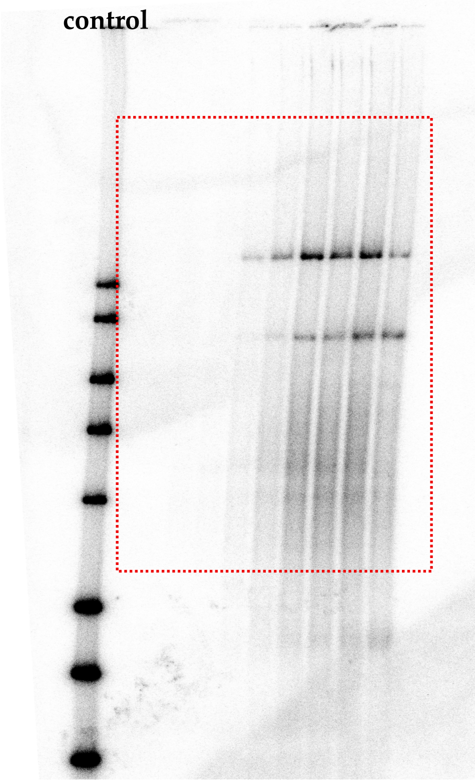

Figure 2 (a)

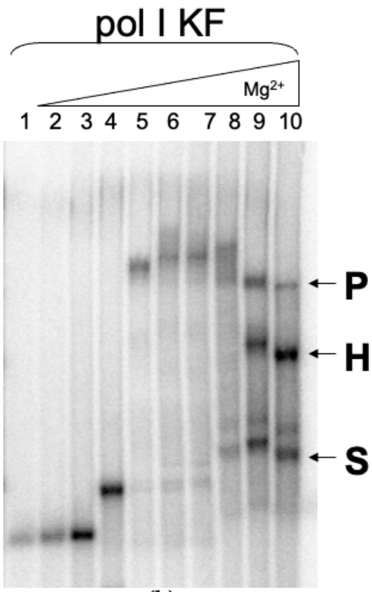

(b)

Uncropped gel

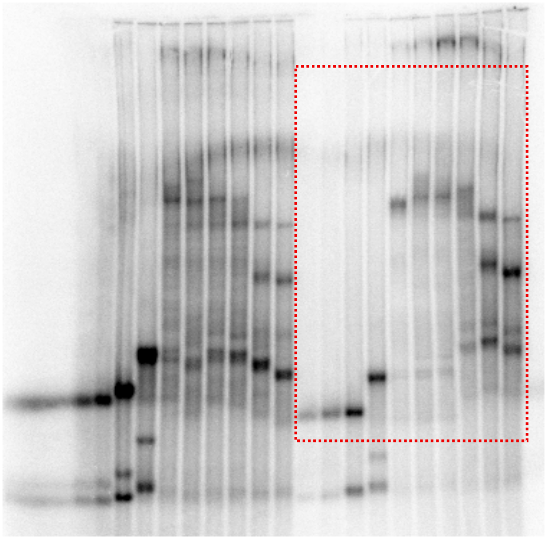

Figure 2 (b)

Magnesium concentration modulates replication slippage of mesophilic and thermophilic DNA polymerases in vitro Castillo-Lizardo, Melissa and Enrique Viguera. Universidad de Málaga.

Figure 3. Effect of magnesium concentration on slippage of DNA polymerase pol I klenow fragment exo$^-$.

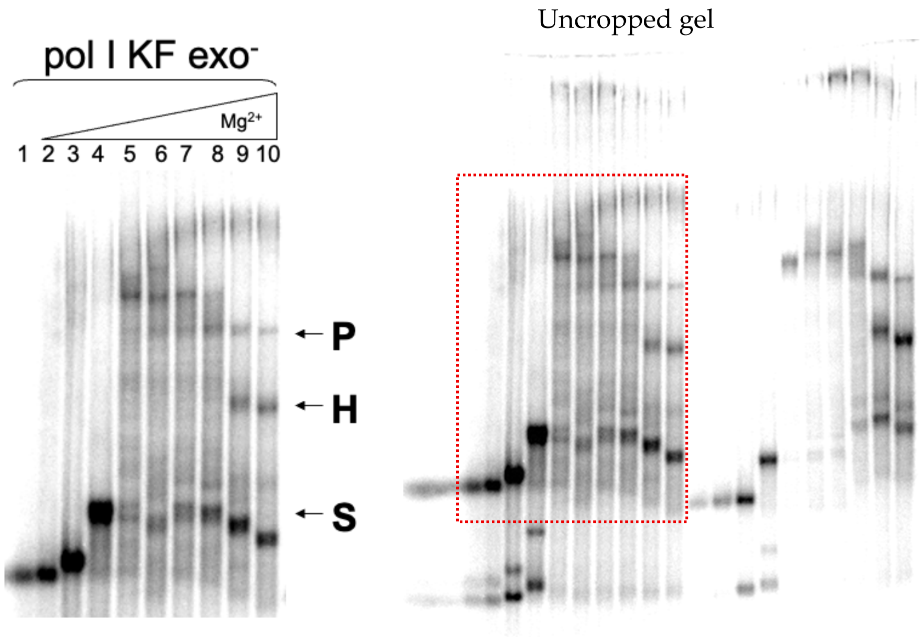

Figure 3.

Figure 4. (a) Effect of magnesium concentration on slippage of *Taq* DNA polymerase. (b) Effect of magnesium concentration on slippage of *Pfu* DNA polymerase.

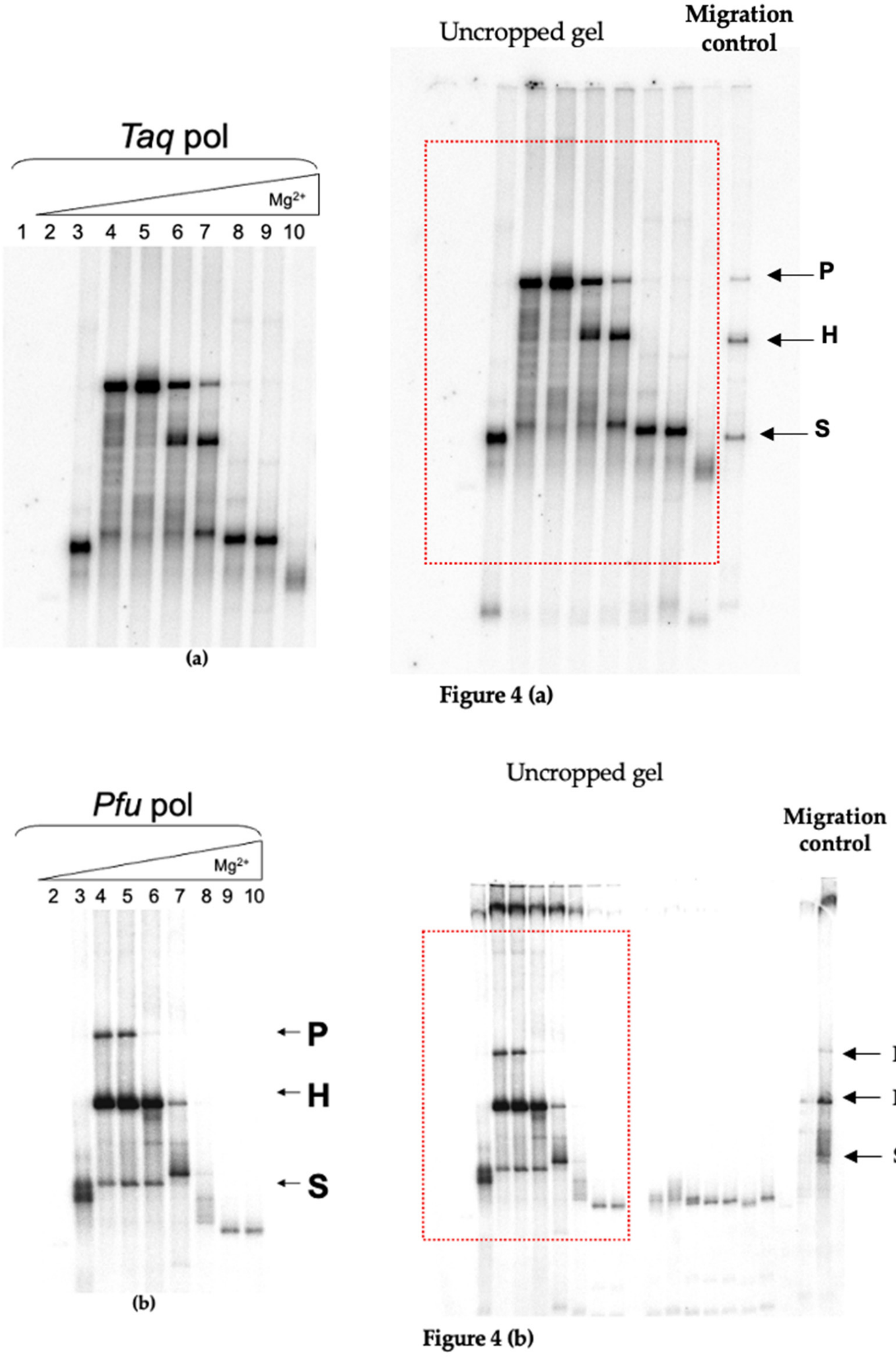

Figure 5. (a) Effect of magnesium concentration on strand displacement activity of DNA polymerases T4, (b) T7 and (c) pol I klenow fragment.

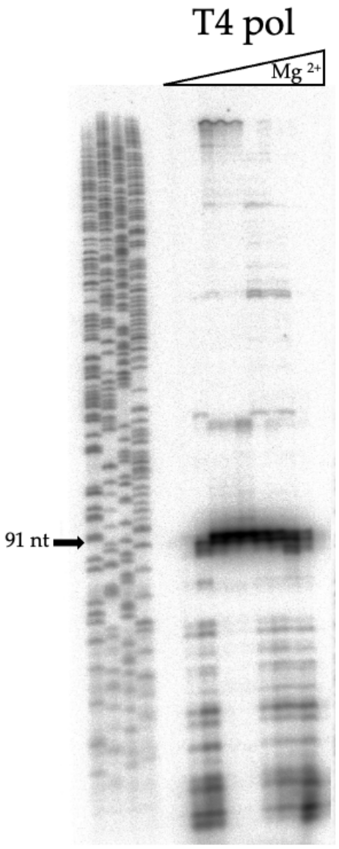

(a)

Uncropped gel

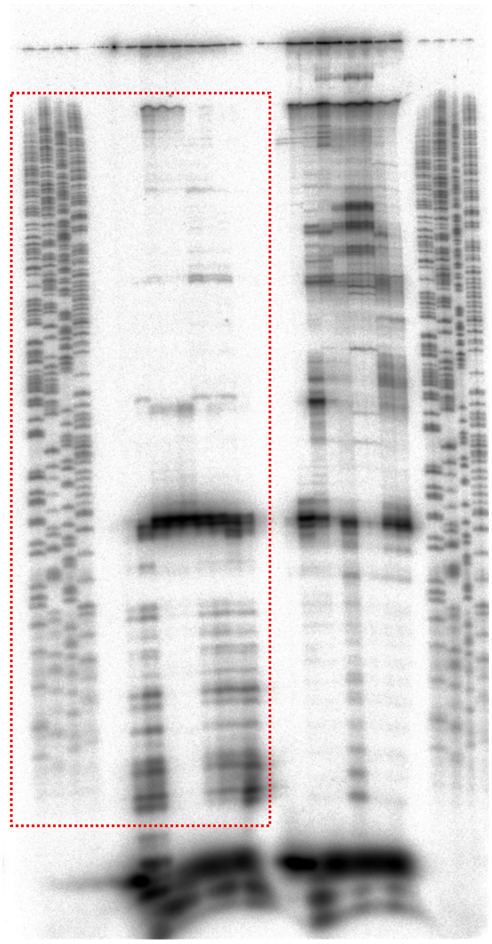

Figure 5. (a)

Uncropped gel

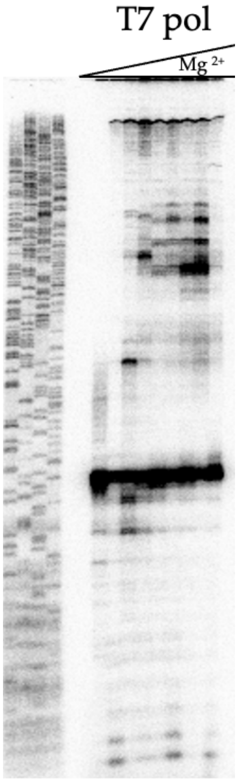

(b)

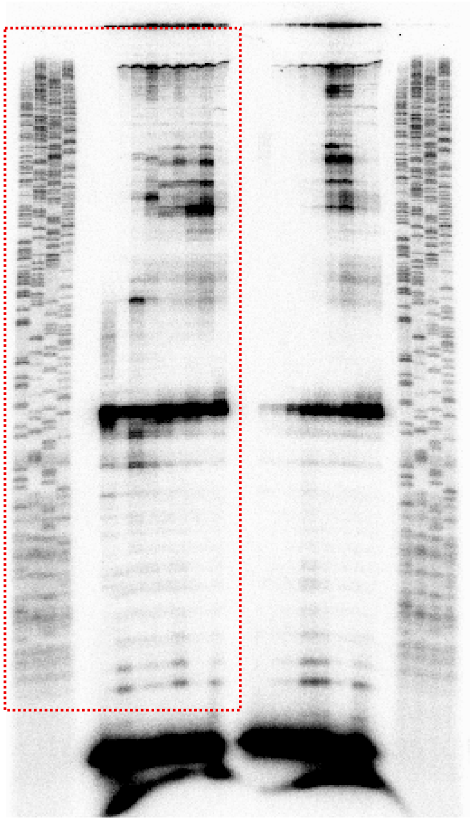

Figure 5. (b)

Magnesium concentration modulates replication slippage of mesophilic and thermophilic DNA polymerases in vitro Castillo-Lizardo, Melissa and Enrique Viguera. Universidad de Málaga.

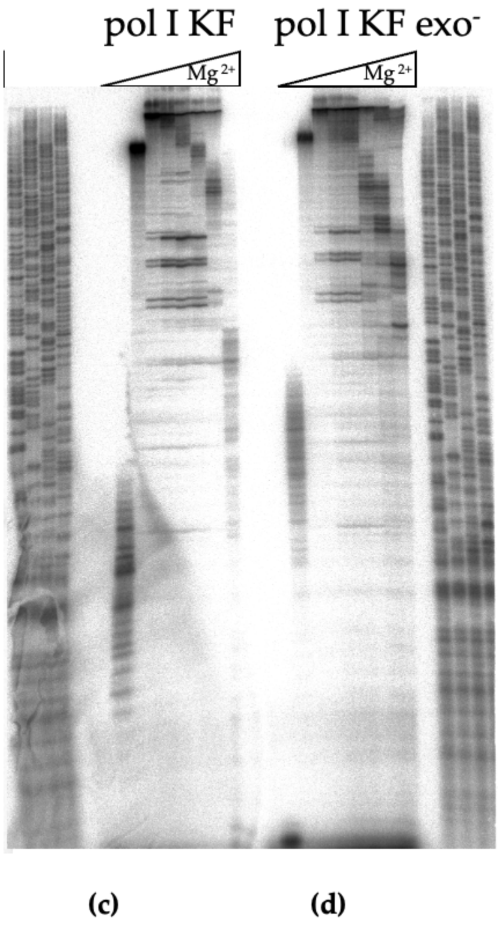

Uncropped gel

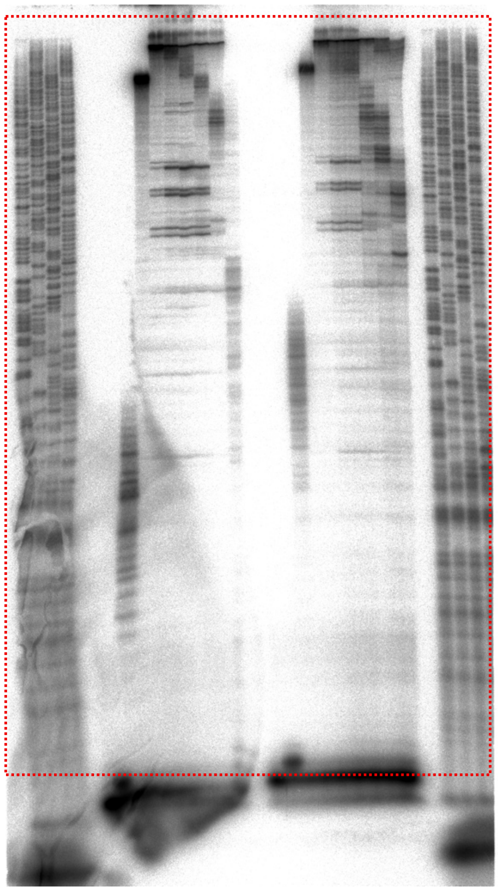

Figures 5. (c), (d)

Figure 6. (a) Effect of magnesium concentration on strand displacement activity of *Pfu* and (b) *Taq* DNA polymerases.

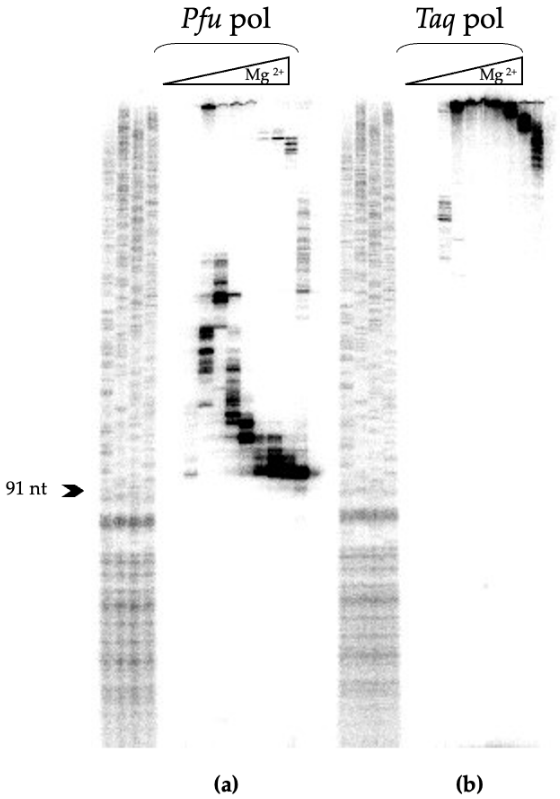

Uncropped gel

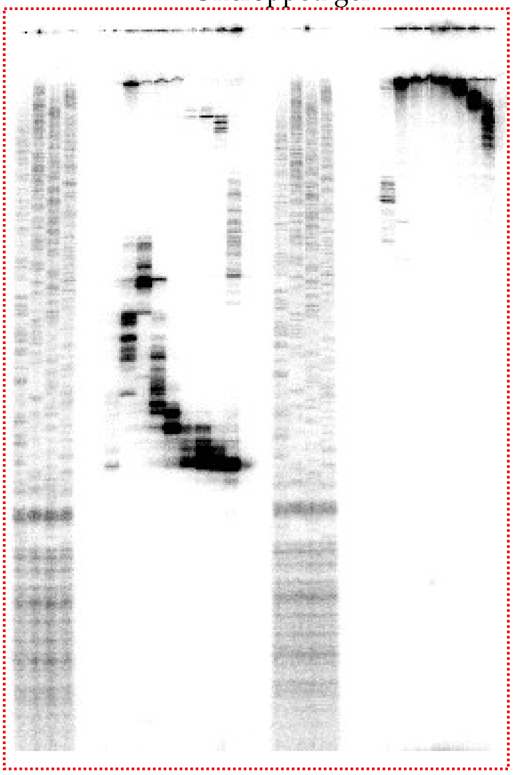

Figures 6 (a), (b)

Magnesium concentration modulates replication slippage of mesophilic and thermophilic DNA polymerases in vitro Castillo-Lizardo, Melissa and Enrique Viguera. Universidad de Málaga.

Uncropped gel

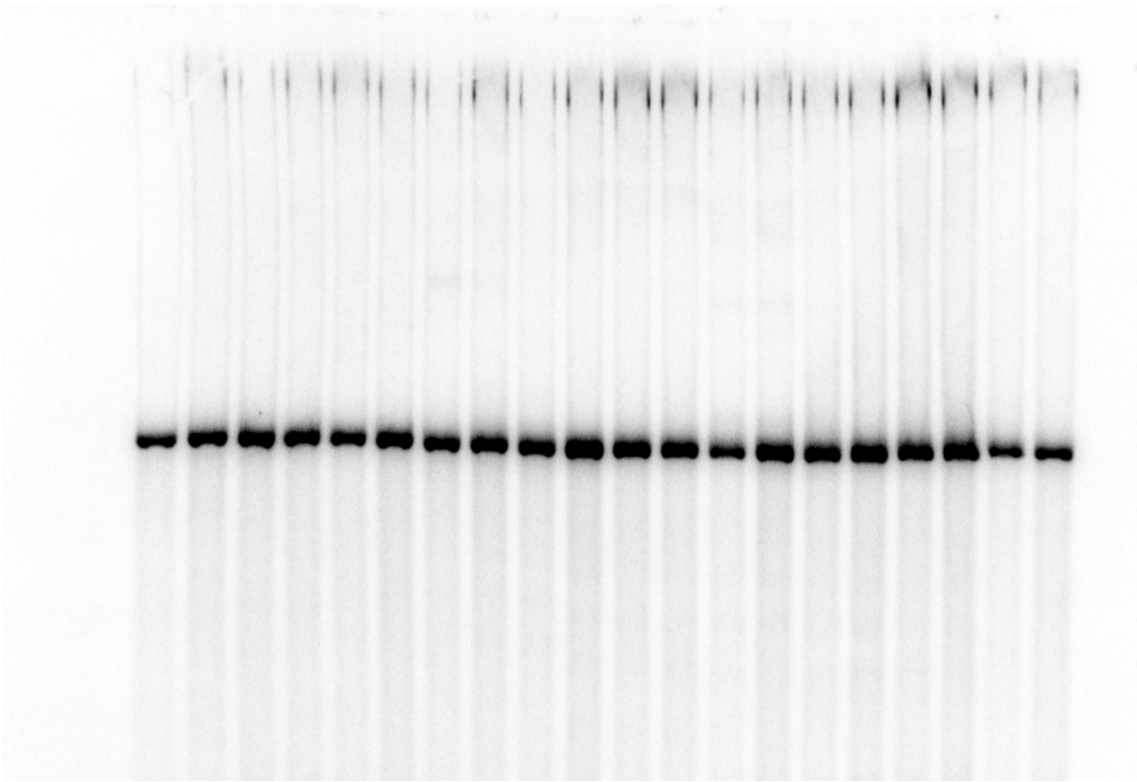

Figure S1

Magnesium concentration modulates replication slippage of mesophilic and thermophilic DNA polymerases in vitro Castillo-Lizardo, Melissa and Enrique Viguera. Universidad de Málaga.
